# First German Academy for Further Medical Training on Rare Diseases (*FAKSE*, http://www.fakse.info)

**DOI:** 10.1186/1750-1172-7-S2-A41

**Published:** 2012-11-22

**Authors:** Julia Giehl, Holm Graessner, Olaf Riess

**Affiliations:** 1 University Hospital of Tuebingen, Department for Medical Genetics, FAKSE, Tuebingen, 72076, Germany

## Problem

On average it will take up to seven years to be diagnosed with an orphan disease. During this time, patients will have seen several doctors, received a large number of changing diagnoses, consulted the internet various times and will have undergone many forms of treatment. Since the majority of rare disorders affect more than one organ system, it is almost impossible for physicians not specialising in rare disorders or working in this area on a daily basis to diagnose an orphan disease.

## Approach

The Centre for Rare Diseases Tuebingen (University Hospital Tuebingen) opened the first German Academy for Further Medical Training on Rare Diseases (*FAKSE*) in April 2011. The goals of the academy are (i) educate practice-based physicians and clinicians on the matter of rare diseases in an interdisciplinary and illustrative fashion, (ii) raise awareness for these disorders and provide physicians with methodologies and “Red Flags” for better recognition of RD and (iii) to bring physicians in contact with relevant experts and patient organisations.

## Methodology

*FAKSE* offers practitioners and clinicians the possibility to receive first-hand information on orphan diseases from interdisciplinary experts. Each training course focuses on a group of related RD such as rare storage disorders and comprises high standard video based lectures, presentation of specific ‘Red Flags’ (symptoms one should think of a rare disorder), and meet-the-experts workshops to discuss unclear cases. Throughout its first year, *FAKSE* organised four training courses and has already trained 250 physicians.

By date, training included courses on rare neurological diseases, rare storage disorders, rare female genital malformations, rare skin disorders, rare infantile malformations of the maxillofacial region and rare eye disorders. In 2012, two more courses on rare auto inflammatory diseases and rare tumours will be held. Further courses for 2013 are already being planned.

Since December 2011, *FAKSE* collaborates with Germany’s umbrella association for patient organisations (ACHSE).

